# The History and Capabilities of Herbal Simples

**Published:** 1890-09-27

**Authors:** W. T. Fernie


### The History and Capabilities of Herbal Simples.

By W. T. Fernie, M.D.

*(Continued from page 373.)*

### XIX.—THE HORSE CHESTNUT.

This is the fruit of a well-known handsome tree which suggests at once fond memories of Bushey Park and of halcyon days spent among its avenues. The large floral spikes which adorn the chestnut groves there in early spring are of soft, creamy velvet, dashed with bright pink. The tree itself hails from the mountains of Northern Asia, and was first brought to England in 1633. Its wood, which is white and soft, has obtained a reputation for making excellent wine casks:—

"The close-grained chestnut, wood of sovereign use
For casking up the grape's most powerful juice."

The nuts are starchy, and contain so much potash that they may be used when boiled for washing purposes. A small stock of them would have been very handy for Peter Simple, at Sally Port, when accosted by the young lady, nicely dressed, with the odd question, "Well, Reefer, how are you off for soap?" In France and Switzerland the nuts are employed for cleansing wool and bleaching linen. Remarkably curative effects are claimed by a special school of modern physicians for a tincture made from these nuts with spirit of wine, as well as for the nuts themselves finely powdered. The sphere acted upon is that of the rectum, or lowest bowel, in cases of piles and obstinate constipation, particularly whenever the bottom of the back gives out on walking, with aching and a sense of weariness in that region. Likewise signal relief is found to be wrought by this remedy when the throat is duskily red and dry, in conjunction with costiveness and piles. Botanically, the horse-chestnut tree is called Æsculus Hippocastanea, from *esca*, food; *hippos*, a horse; and the city—Castana. By some it has been supposed that the term "horse-chestnut" has been applied because this fruit has proved useful in the pulmonary diseases of horses. The tree was received by us from Turkey, where its nuts are used for horses that are "broken, or touched in the wind." But in this country horses will not eat them. Also it has been ingeniously suggested that the cicatrix of the leaf resembles a horseshoe, with all its nails evenly placed. But much more probably the epithet implies size and coarseness by comparison with the sweet Spanish chestnut. In the same way we talk of the horse-radish, the horse-daisy, and (Henry V., Act 2, Sc. 4), the horse-leech. Sir Thomas Browne remarked long since, in his "Vulgar Errors," "in the names of the horse-mint, the bulrush, and many more, the expression is but a græcism by the prefix of horse, and bull—*hippos*, and *bous*—intending no more than great." From the leaves of the horse-chestnut an extract is made which has proved of service in curing whooping-cough, and of which from one-third to half a teaspoonful may be given for a dose. According to old folk lore a chestnut begged or stolen was supposed to be preservative against rheumatism. If it be powdered and sniffed up the nostrils, a small portion will excite active sneezing, for which purpose the Edinburgh College of Medicine introduced it into their pharmacopœia. Medicinally, the ripe nut is employed, deprived of its shell, and collected in September or October. On the Continent the bark of the horse-chestnut tree is held in estimation as a febrifuge, and is thought to act in the same way as Peruvian bark, but it is much more astringent. Here the rich, glossy, red-brown nuts have given their name to a certain shade of mellow dark auburn hair. Rosalind, in *As You Like It*, says, Orlando's locks are of a good colour.

"I'faith, your chestnut was ever the only colour." It was probably about the fruit of the Spanish tree Shakespeare said "A woman's tongue gives not half so great a blow to the ear as will a chestnut in a farmer's fire." Taken altogether, the horse-chestnut for its splendour of blossom and wealth of umbrageous leaf, its polished mahogany fruit, and its special medicinal virtues, is, *facile princeps*, the belle of our trees. But, like many a ball-room beauty, when the time comes for putting aside the gay attire, it is sadly untidy and makes a great litter.

